# Supplementary material for: Atmospheric isoprene measurements reveal larger-than-expected Southern Ocean emissions
Source: Nat Commun. 2024 Mar 22;15:2571. doi: 10.1038/s41467-024-46744-4 (PMC10959939; doi:10.1038/s41467-024-46744-4)
Supplement: Supplementary file 1 — Supplementary Information [file 41467_2024_46744_MOESM1_ESM.pdf]

Supplementary Information to:

Atmospheric isoprene measurements reveal larger-than-expected Southern Ocean emissions

Valerio Ferracci[1 + * ∞], James Weber[2 ♦ * ∞], Conor G. Bolas[3 #], Andrew D. Robinson [3 §], Fiona Tummon[4], Pablo Rodríguez-Ros[5 @], Pau Cortés-Greus[5], Andrea Baccarini,[6 ¶] Roderic L. Jones[3], Martí Galí[5], Rafel Simó[5], Julia Schmale[6], Neil. R.P. Harris[1]

[1] Cranfield Environment Centre, Cranfield University, College Road, Cranfield, MK43 0AL, UK

[2] School of Biosciences, University of Sheffield, Sheffield, S10 2TN, UK

[3] Department of Chemistry, University of Cambridge, Lensfield Road, Cambridge, CB2 1EW, UK

[4] Swiss Federal Office for Meteorology and Climatology MeteoSwiss, Payerne, Switzerland

[5] Institut de Ciències del Mar (ICM-CSIC), Barcelona, Catalonia, Spain

[6] Extreme Environments Research Laboratory, École Polytechnique Fédérale de Lausanne, Switzerland

* these authors contributed equally to this work

[+] now at National Physical Laboratory, Hampton Road, Teddington TW11 0LW, UK

[♦] now at Dept of Meteorology, University of Reading, Reading RG6 6BB, UK

[#] now at ITOPF, Old Broad Street, London EC2M 1QS

[§] now at Schlumberger Cambridge Research, Madingley Road, Cambridge, CB3 0EL, UK

[@] now at Marilles Foundation, Bisbe Perelló, Palma, Mallorca, Spain

[¶] now at Laboratory of atmospheric processes and their impact, École Polytechnique Fédérale de Lausanne, Switzerland

[∞] corresponding authors. Email: v.ferracci@cranfield.ac.uk; j.m.weber@reading.ac.uk

Supplementary Figures

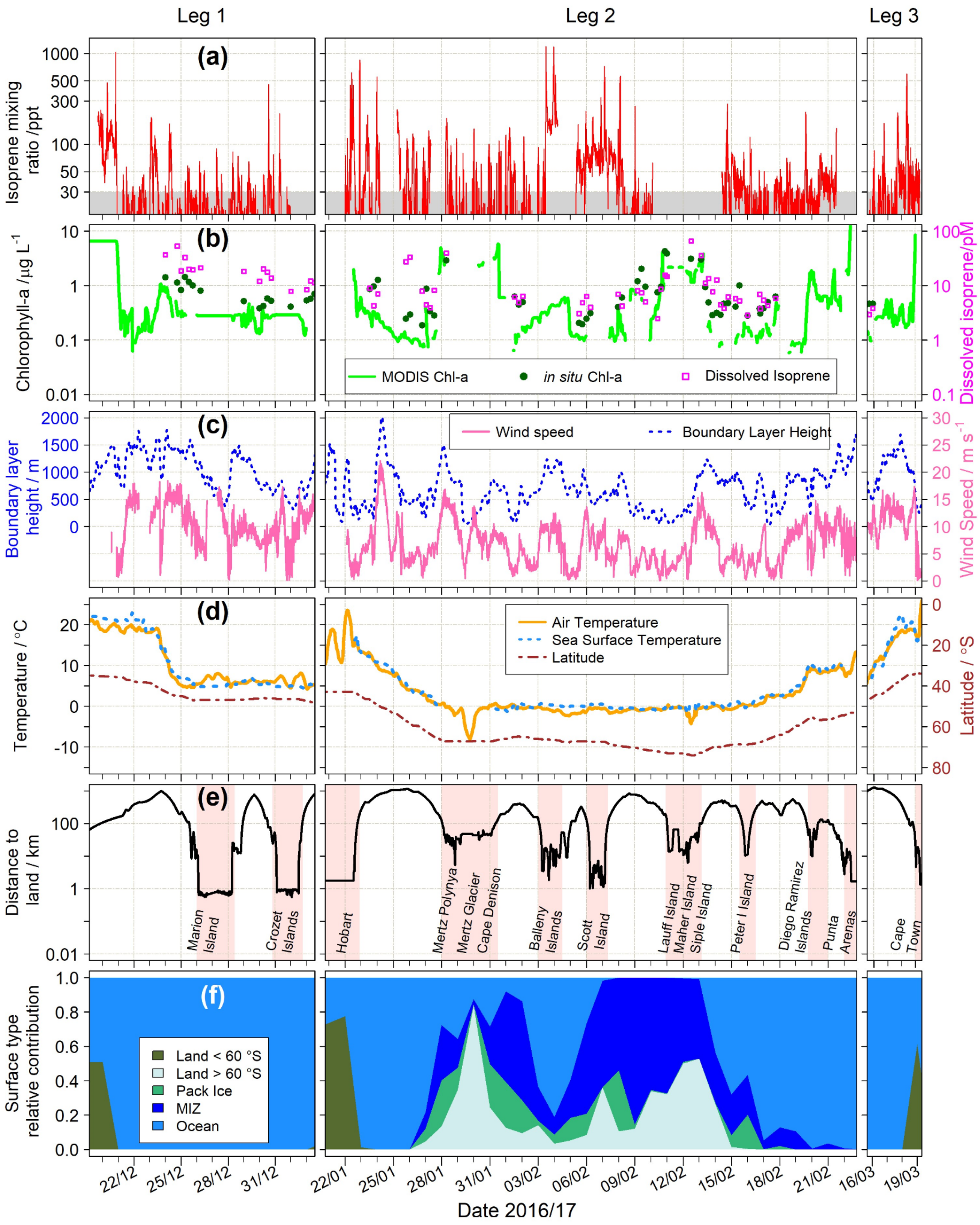

Fig S1: Time series of selected variables during ACE: (a) isoprene mixing ratios, with the grey shaded area indicating data below the LOD; (b) chlorophyll-a and dissolved isoprene from in situ measurements along with chlorophyll-a from satellite retrievals (MODIS-Aqua); (c) boundary layer height and wind speed; (d) air and sea surface temperature along with latitude; (e)

distance to land: periods of time in which the ship was within ~100 m of land are shown as pink-shaded areas, along with the toponyms on the bottom of the panel; (f) daily mean relative contribution of surface types along the air mass back trajectory. Note that a logarithmic scale was used for the y axis in (a), (b), (e).

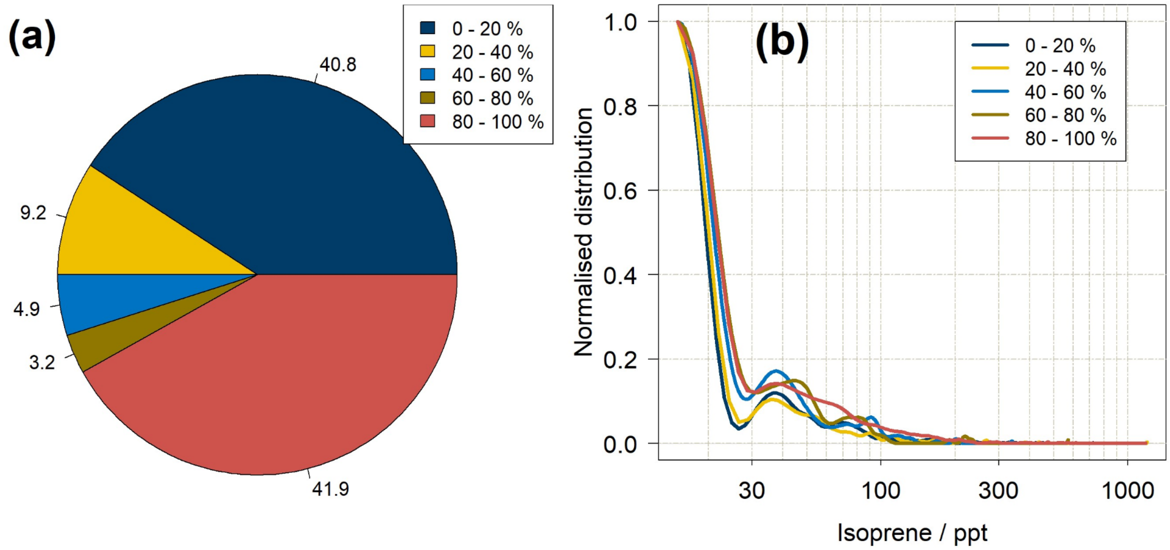

Fig S2: Analysis of the impact of ship exhaust on observed ambient isoprene. (a) Percentage contribution from each of the five exhaust bins; (b) probability distribution of isoprene data in the five exhaust exposure bins normalised to their highest value.

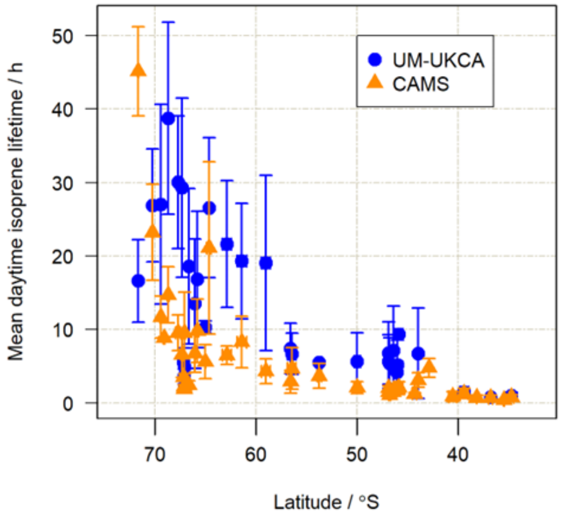

Fig S3. Mean daytime (10:00-15:00 local solar time) lifetime of isoprene (in hours) with respect to reactions with OH, NO_3 and O_3 as a function of latitude along the ACE cruise track. Blue circles: OH and NO_3 from UM-UKCA model runs (Ferracci et al., 2018), O_3 and air temperature (used for rate coefficient calculation from Arrhenius expressions) from *in situ* measurements. Orange triangles: all data from CAMS model (Inness et al., 2019). The error bars correspond to 1 standard deviation in isoprene lifetime and latitude over 10:00-15:00 for each day.

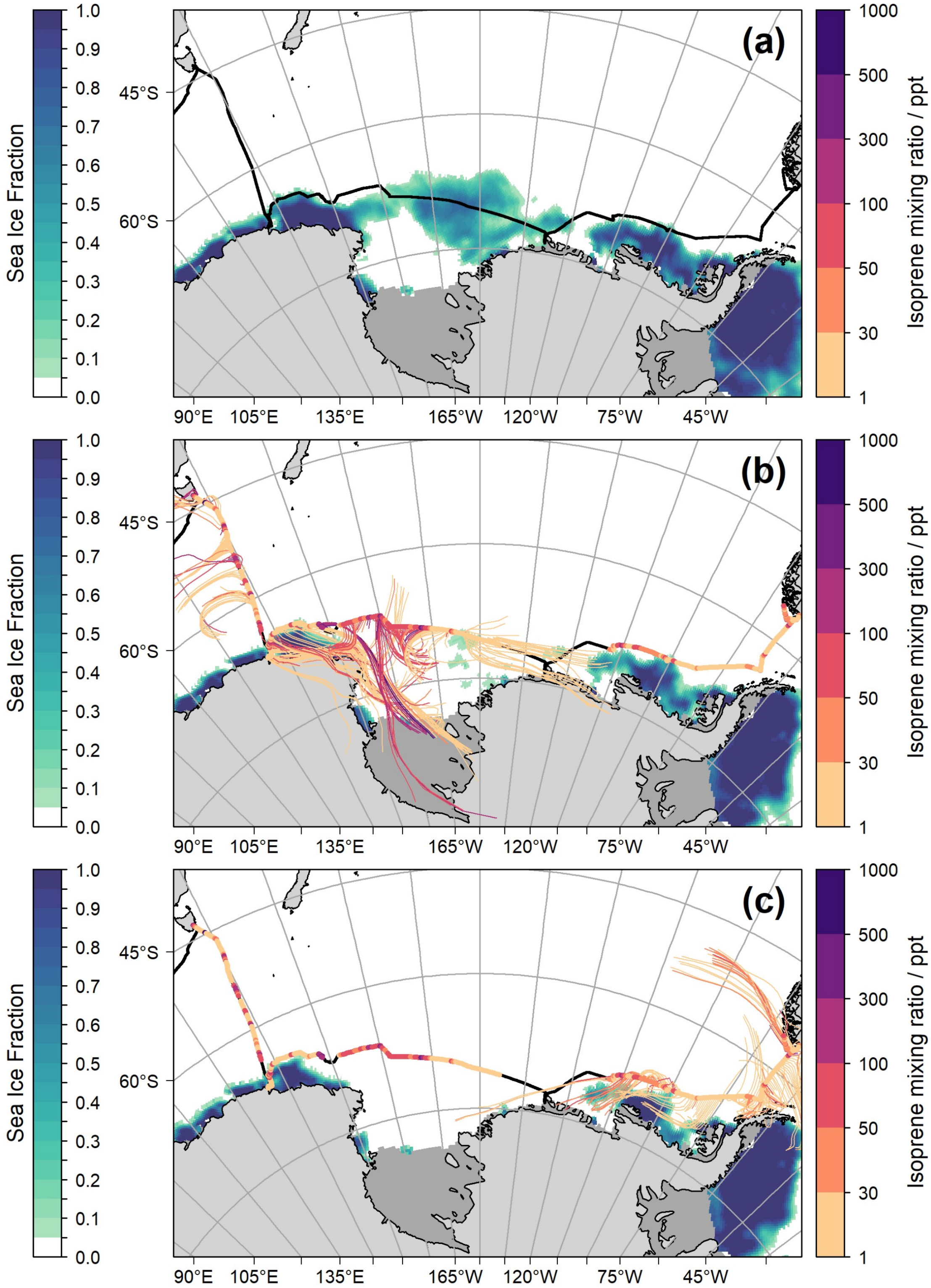

Fig S4: Maps of average sea ice fraction (strip legend on the left) before and during Leg 2 of ACE, for the periods (a) 1 - 19 Jan 2017, (b) 20 Jan - 9 Feb 2017 and (c) 10 - 22 Feb 2017. The ACE track is shown as a black solid line, with overlaid isoprene mixing ratios (in units of ppb, or nmol mol^{-1}, strip legend on the right). The back-trajectories, shown as thin coloured lines, are adjusted for the isoprene lifetime at each point along the cruise track, with a maximum allowed

lifetime of 48 h. The colour of each back-trajectory reflects the isoprene mixing ratio at the point along the cruise track from which it originates. Ice shelves are shown as dark grey areas.

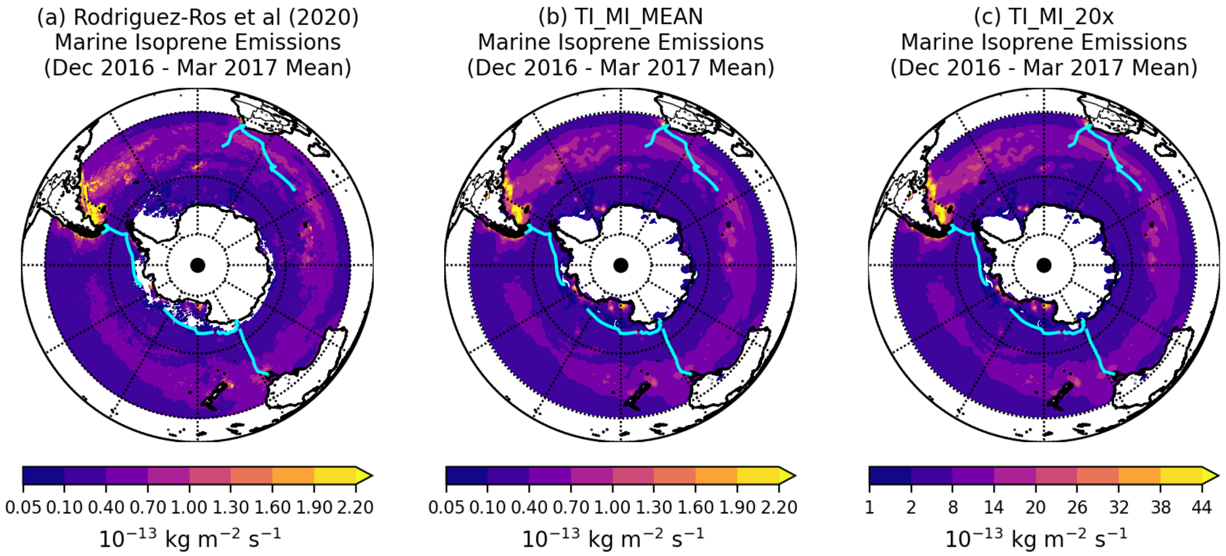

Fig S5. Marine isoprene emissions averaged over December 2016 - March 2017 (inclusive) from (a) Rodriguez-Ros et al (2020), (b) as used here for TI_MI_MEAN and (c) as used here for TI_MI_20x (note different colour scale for (c)).

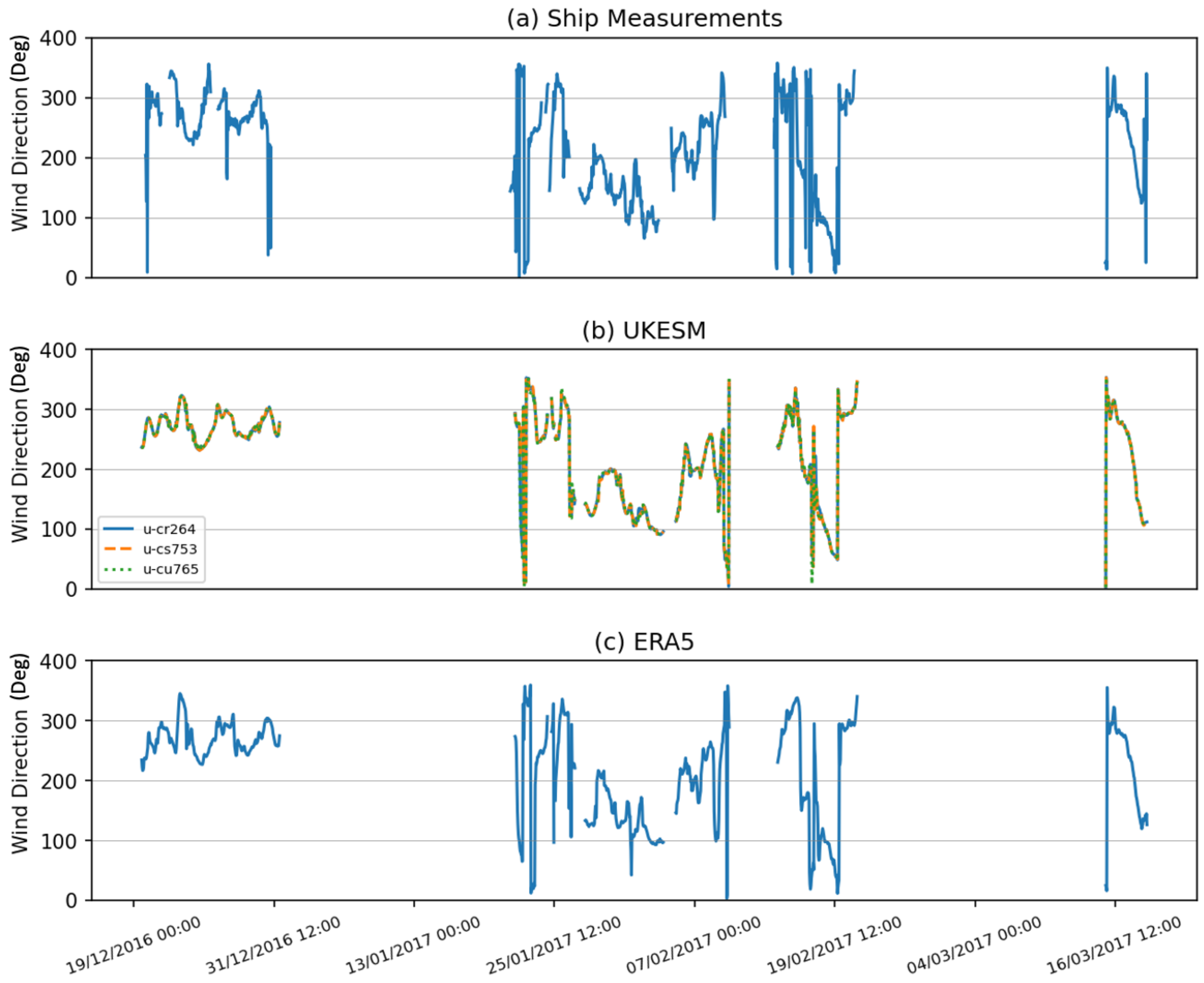

Fig S6. Wind direction (a) measured by ACE ship, (b) simulated by UKESM1 in model grid cell containing ship at the relevant time and (c) from ERA5 reanalysis in ERA5 grid cell containing ship at the relevant time. In (b) the u-xxxxx term refers to specific model runs in UKESM.

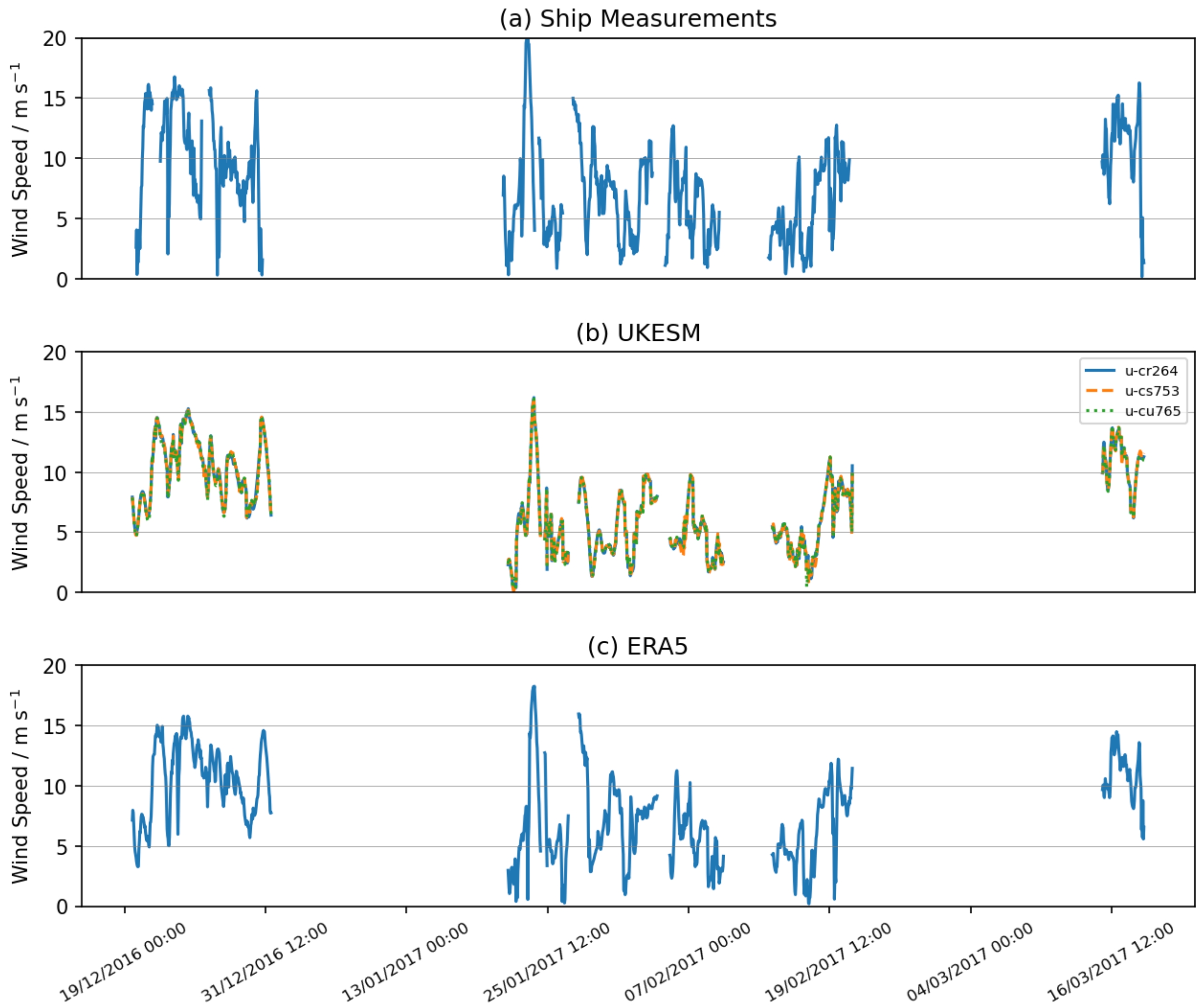

Fig S7. Wind speed (a) measured by ACE ship, (b) simulated by UKESM1 in model grid cell containing ship at the relevant time and (c) from ERA5 reanalysis in ERA5 grid cell containing ship at the relevant time.

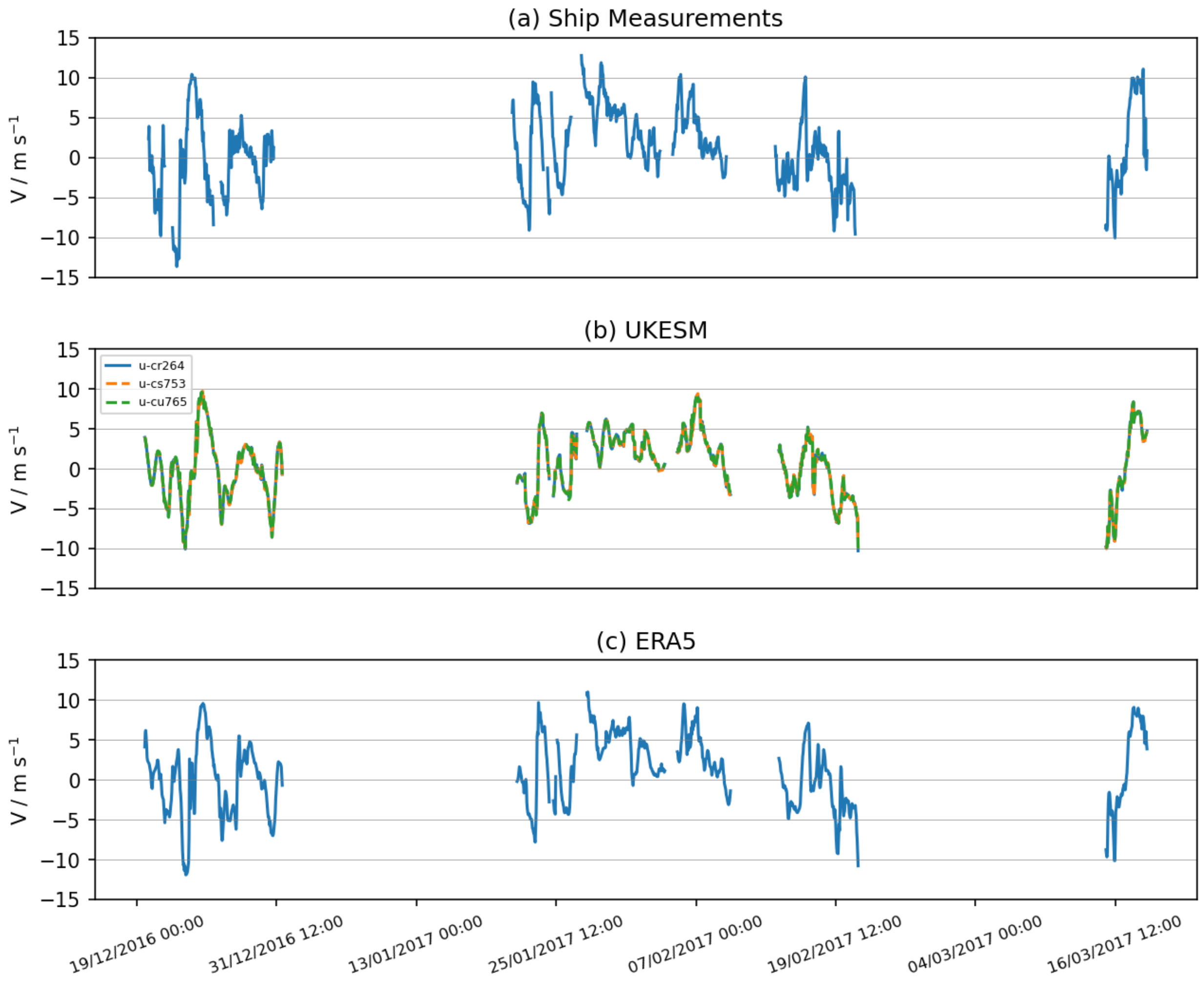

Fig S8. South to North wind component (a) measured by ACE ship, (b) simulated by UKESM1 in model grid cell containing ship at the relevant time and (c) from ERA5 reanalysis in ERA5 grid cell containing ship at the relevant time.

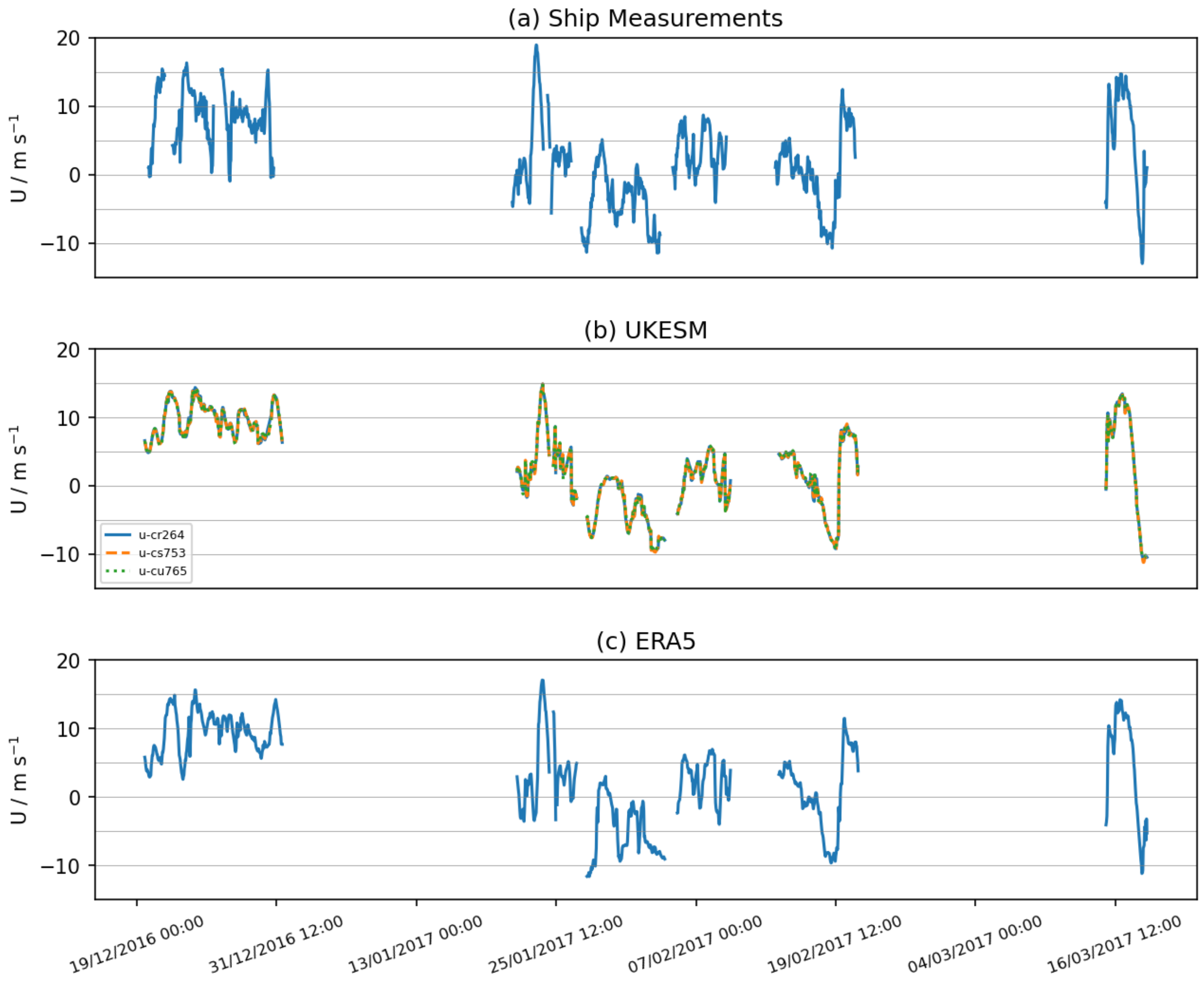

Fig S9. West to East wind component (a) measured by ACE ship, (b) simulated by UKESM1 in model grid cell containing ship at the relevant time and (c) from ERA5 reanalysis in ERA5 grid cell containing ship at the relevant time.

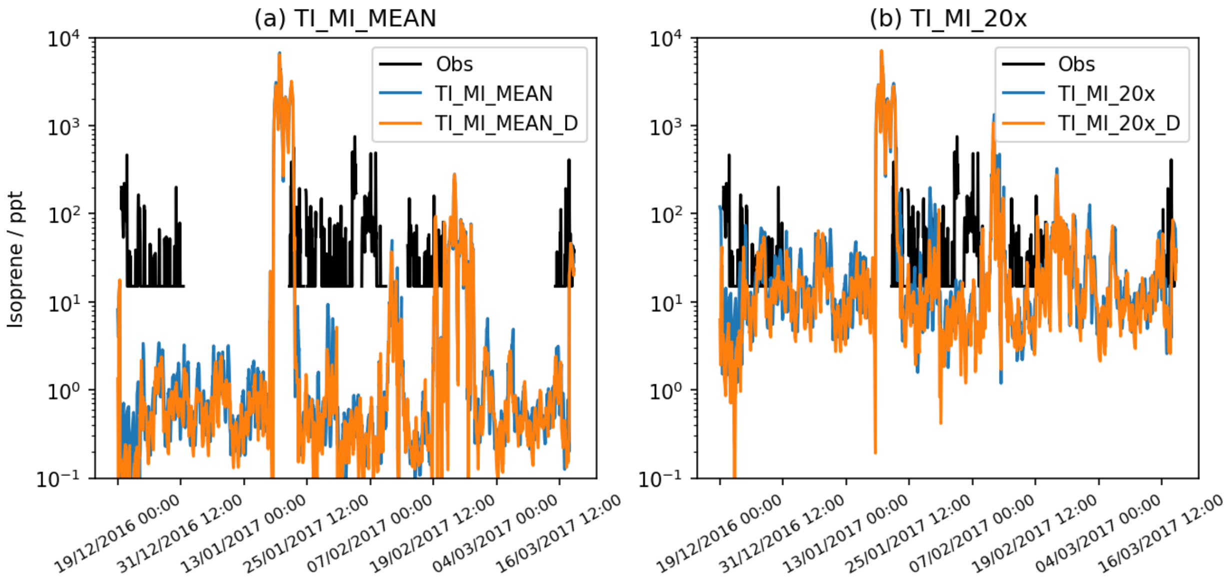

Fig S10. Observed isoprene along ship track and simulated isoprene from (a) TI_MI_MEAN and TI_MI_MEAN_D and (b) TI_MI_20x and TI_MI_20x_D.

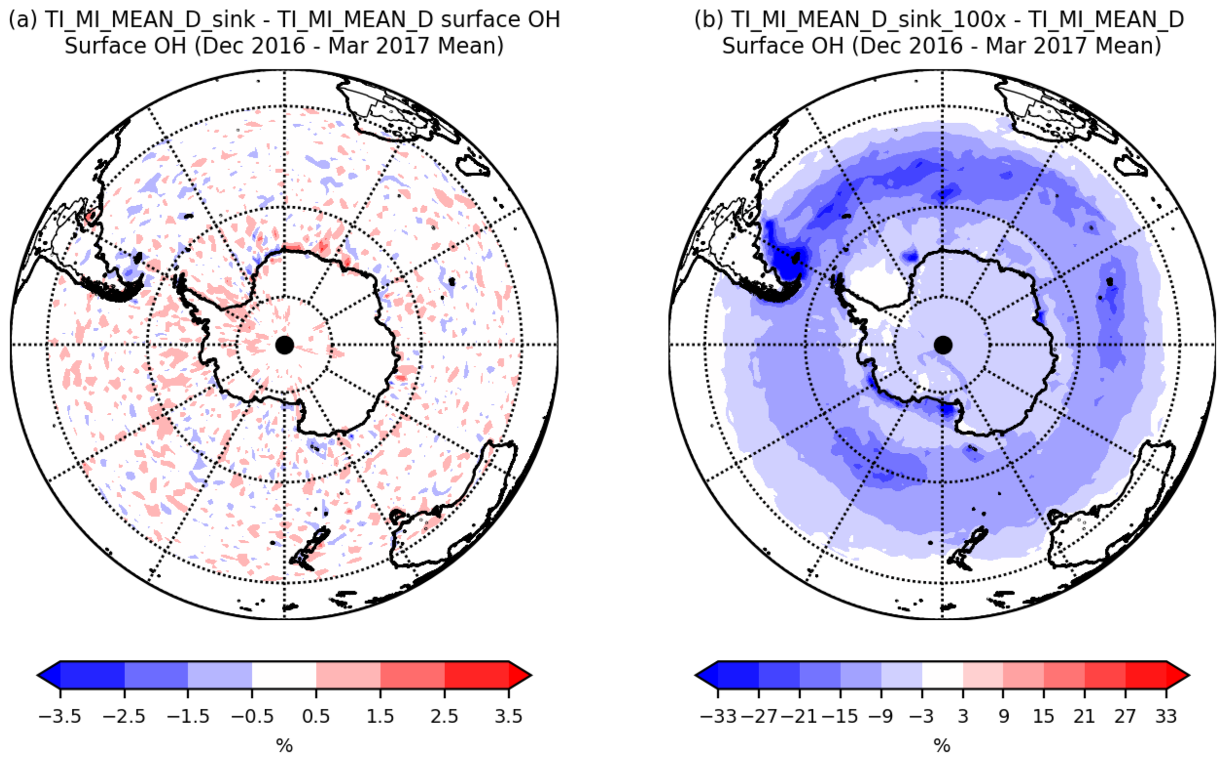

Fig S11. Difference in surface OH averaged over December 2016 - March 2017 (inclusive) between (a) TI_MI_MEAN_D_sink and TI_MI_MEAN_D and (b) TI_MI_MEAN_D_sink_100x and TI_MI_MEAN_D.

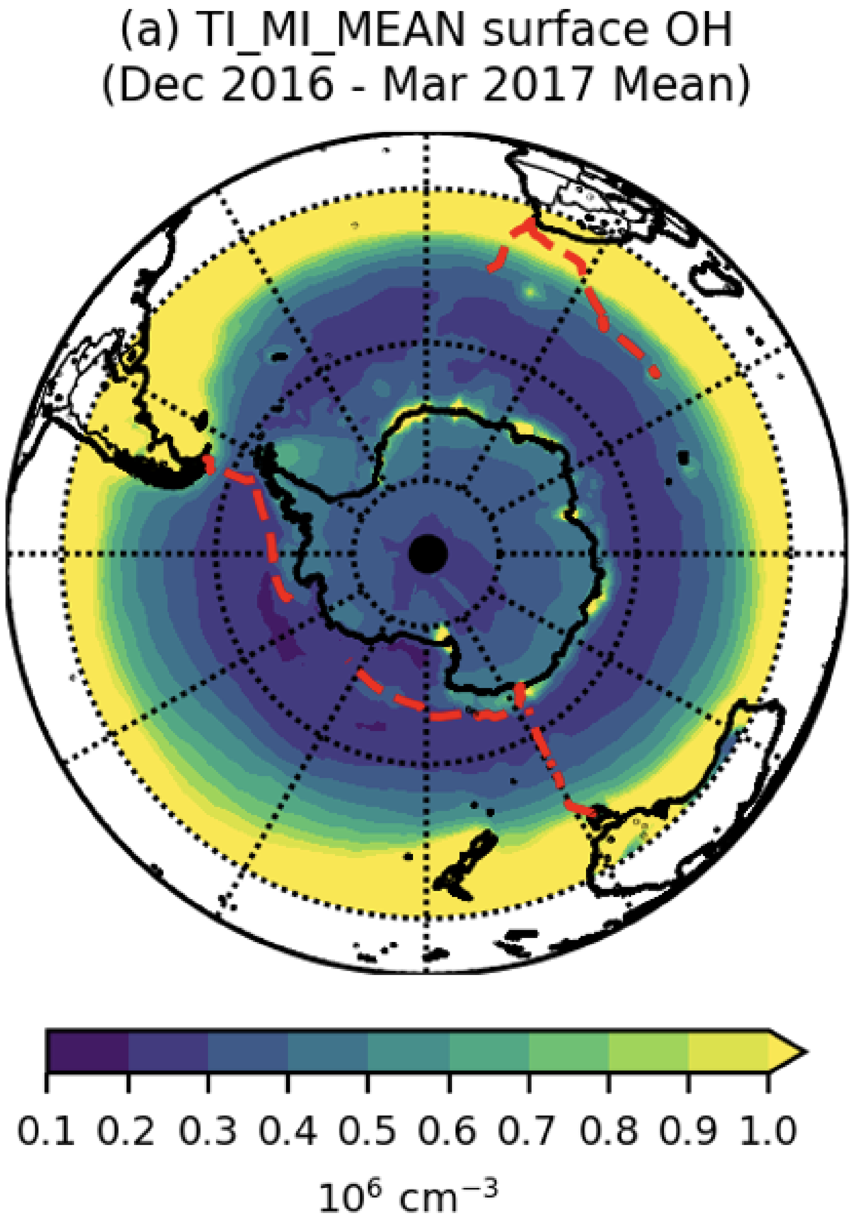

Fig S12. Mean surface OH for Dec 2016 - March 2017 in TI_MI_MEAN/ Red dashed line shows ship track.

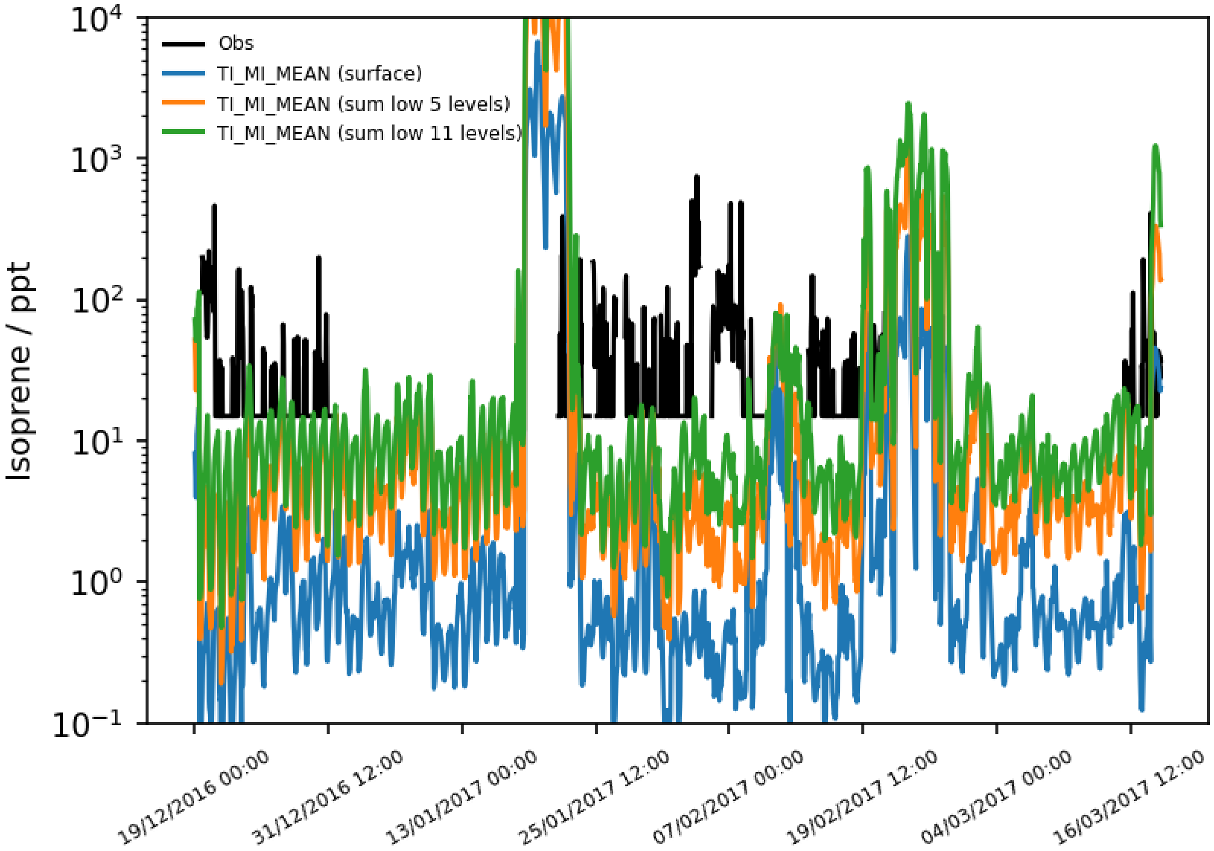

Fig S13. Observed isoprene concentration, isoprene concentration from just the surface level in TI_MI_MEAN and the isoprene concentration if all the isoprene in the lowest 5 models levels (~280 m) and 11 models levels (~1000 m) were compressed into the surface layer.

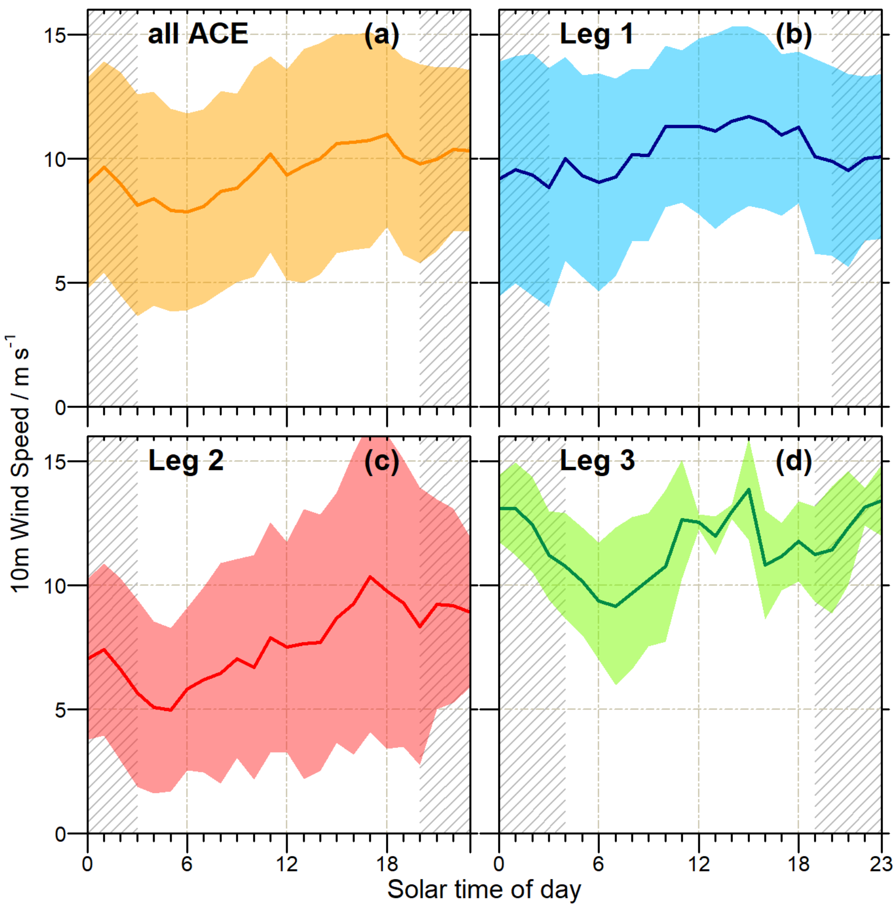

Fig S14. Diel cycle of wind speed during times of marine-originated isoprene for (a) the entire ACE campaign, (b) Leg 1, (c) Leg 2 and (d) Leg 3. Grey-hatched areas indicate nighttime, shaded areas represent 1 standard deviation above and below each 1-h mean.

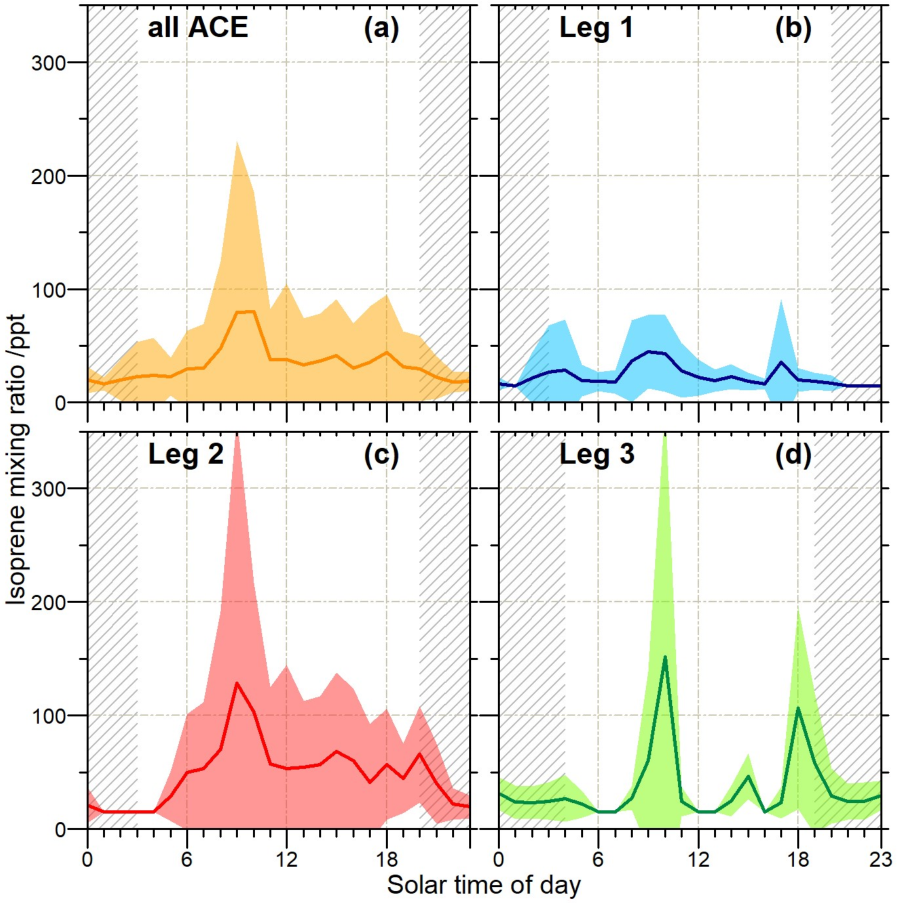

Fig S15. Diel cycle for marine-only isoprene. Concentrations below LOD (30 ppt) were set to half the LOD (15 ppt) after Hackenberg et al. (2017). Grey-hatched areas indicate nighttime, shaded areas represent 1 standard deviation above and below each 1-h mean.

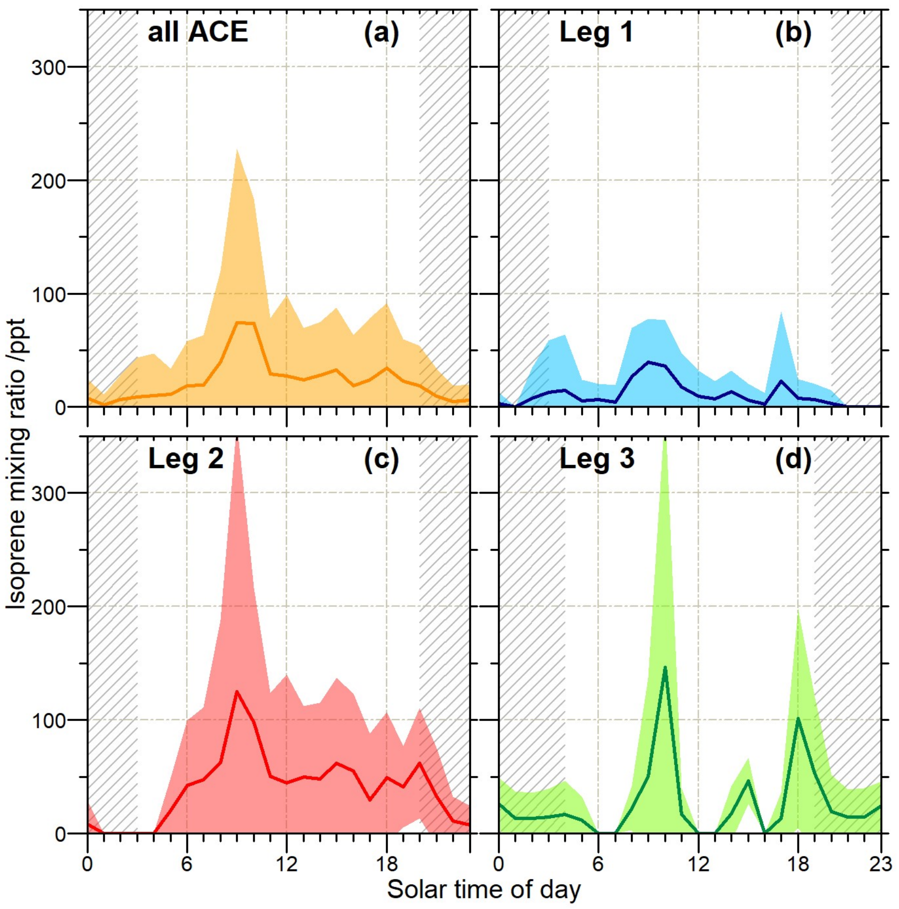

Fig S16. Diel cycle for marine-only isoprene. Concentrations below LOD (30 ppt) were set to zero. Grey-hatched areas indicate nighttime, shaded areas represent 1 standard deviation above and below each 1-h mean.

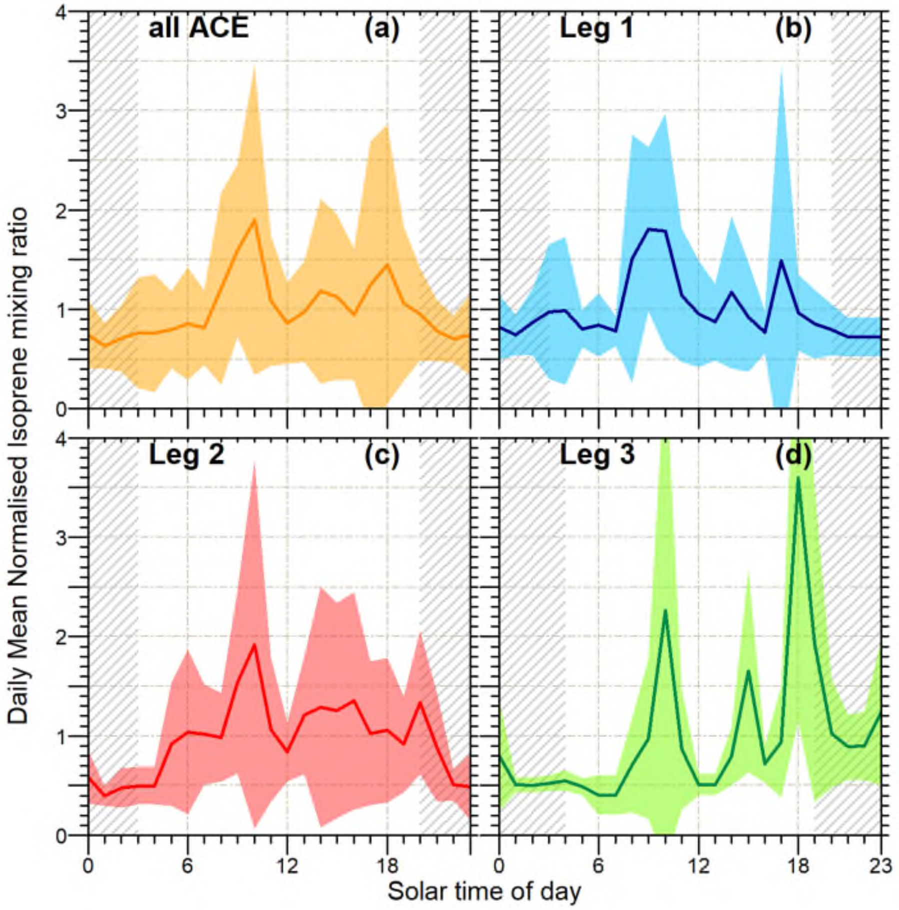

Fig S17. Diel cycle of marine-originated isoprene for (a) the entire ACE campaign, (b) Leg 1, (c) Leg 2 and (d) Leg 3. Values have been normalised to the daily mean isoprene (after Wohl et al., 2020 [4]). Grey-hatched areas indicate nighttime, shaded areas represent 1 standard deviation above and below each 1-h mean.

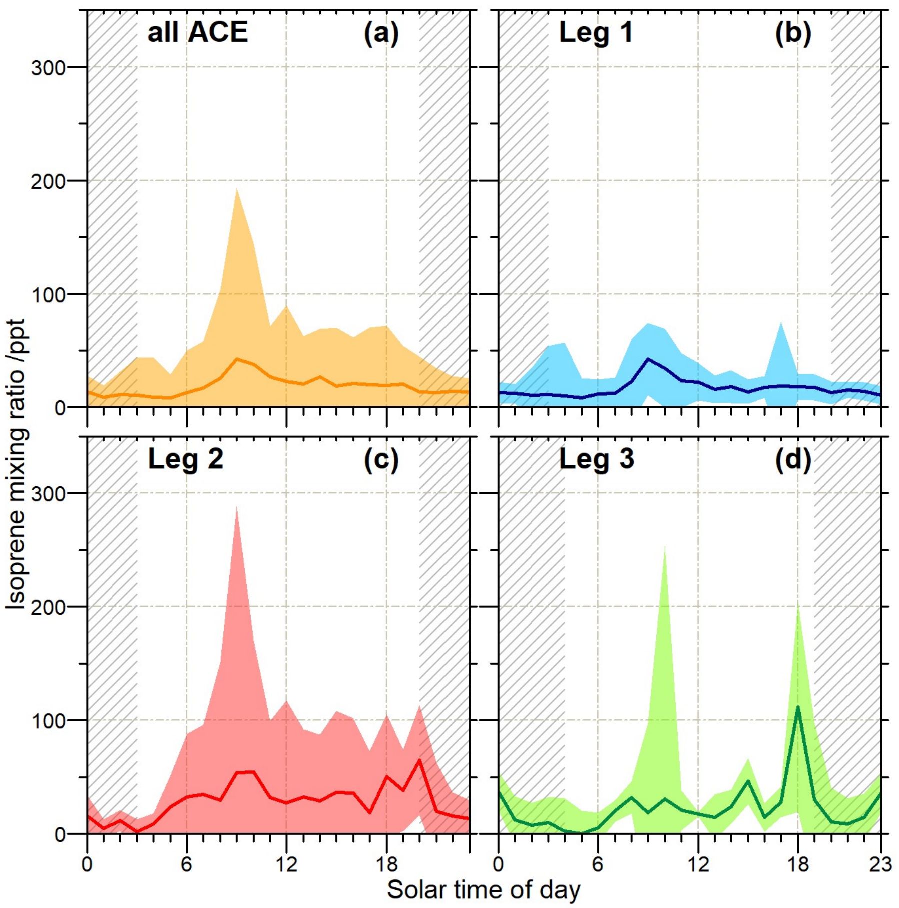

Fig S18. Median diel cycle for marine-only isoprene. Grey-hatched areas indicate nighttime, shaded areas represent 1 standard deviation above and below each 1-h mean.

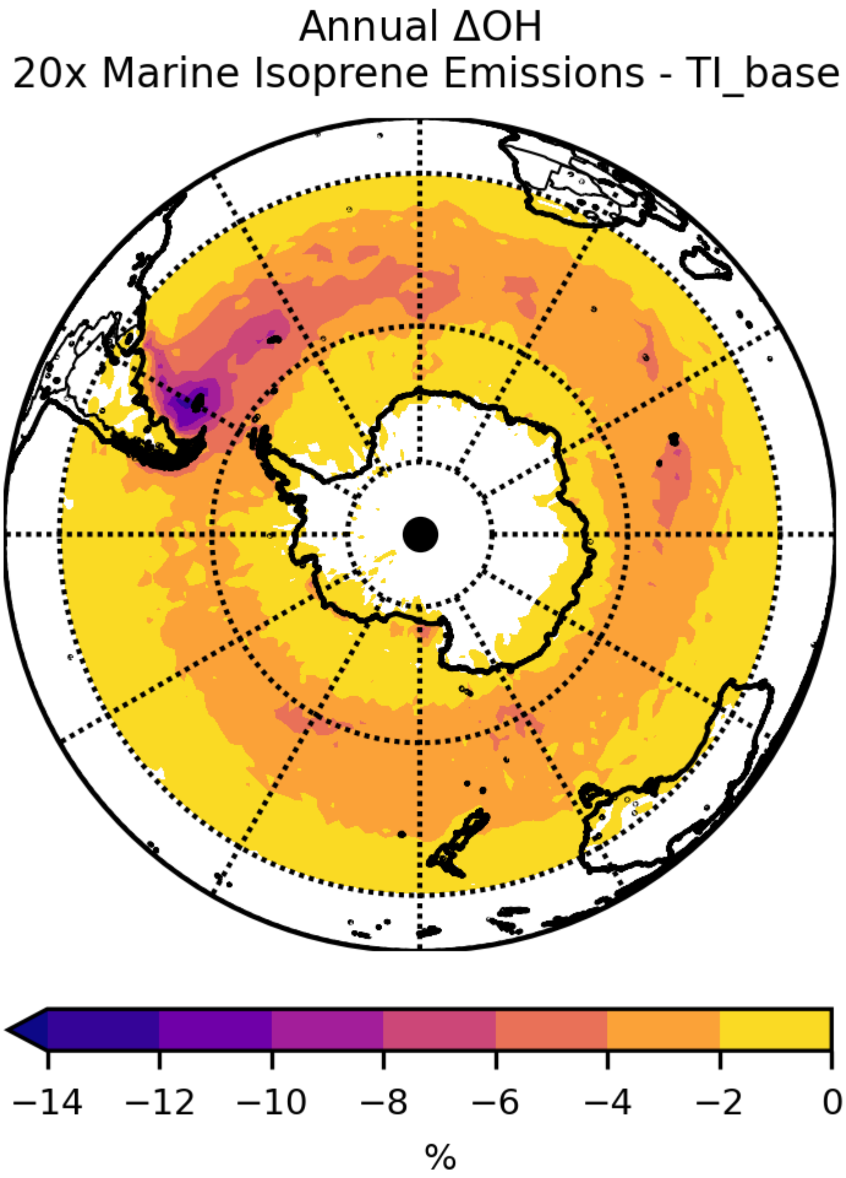

Fig S19. Percentage change in annual mean OH in lowest ~150m between run with 20x marine isoprene emissions and TI_base.

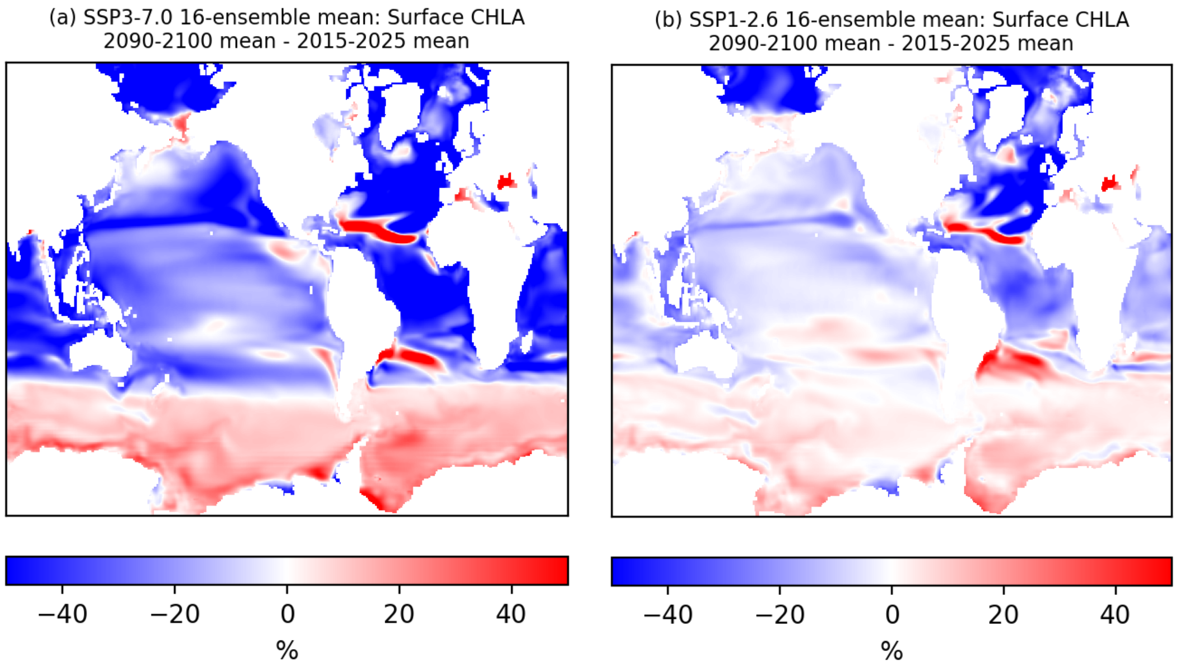

Fig S20. Percentage change in surface chlorophyll concentration between 2015-2025 mean and 2090-2100 mean for (a) SSP3-7.0 and (b) SSP1-2.6. For each time period, an average of 16 ensemble members was taken.

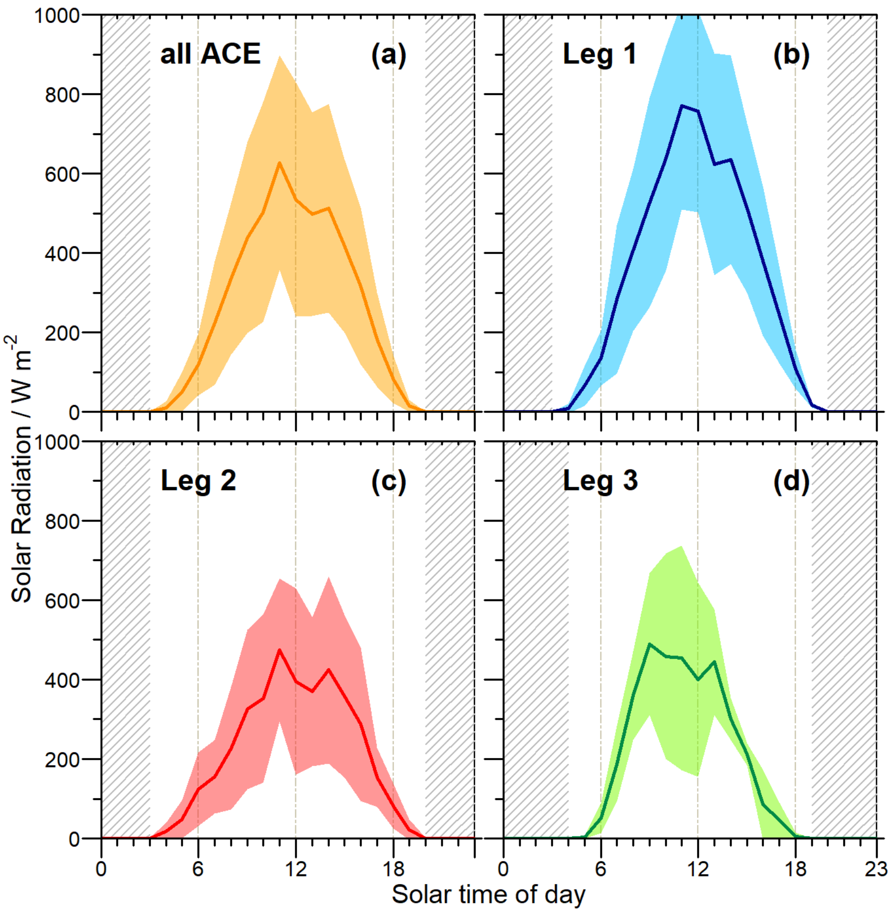

Fig S21. Diel cycle of solar irradiance (SR) during times of marine-originated isoprene for (a) the entire ACE campaign, (b) Leg 1, (c) Leg 2 and (d) Leg 3. Grey-hatched areas indicate nighttime, defined as periods when SR < 1 W m^{-2}, while shaded areas represent 1 standard deviation above and below each 1-h mean

Supplementary Text

Ship exhaust analysis

Measurements during the ACE campaign were at times affected by the vessel's exhaust plume, depending on wind direction, wind speed and vertical atmospheric stability. Previous work developed an exhaust mask using the measurements of CO_2 mixing ratio, black carbon mass concentration and particle number concentrations, which can be applied to filter the measurements [1]. While previous studies reported the presence of isoprene in vehicle exhaust in urban environments [2], there is little information about the isoprene content of ship exhaust. Given the low background levels in the SO, the presence of isoprene in the exhaust, even if minimal, may give rise to high abundances which would be erroneously attributed to biogenic

sources. In addition, conditions under which the sample inlets are affected by the ship exhaust can be thought of as being more generally influenced by emissions from the vessel's footprint (*i.e.*, by emissions from the vessel and its occupants *e.g.*, with ventilation ports acting as point sources). Exhaled breath from humans is a known source of isoprene[3] and, at the low background isoprene levels typical of remote oceans, this could potentially give rise to spikes in the dataset. The exhaust mask used is at a temporal resolution of 1 minute. Given that the typical sampling time of the iDirac during the ACE campaign ranged from 6 to 8 minutes, we calculated a relative contribution of the exhaust to the sampling time of each isoprene measurement: an "exhaust exposure" of 100% indicates that the whole sampling period was affected by the exhaust plume, whereas a value of 0% indicates a sampling period devoid of any influence from the exhaust.

Results of this analysis are shown in Figure S2. The data was grouped in five bins representing exhaust exposures of 0-20%, 20-40%, 40-60%, 60-80% and 80-100%. Interestingly, the majority of the data (> 80%) was in the bins at the two extremes of the exposure scale, as shown in Figure S2a. Upon closer analysis of the distributions of the isoprene mixing ratios in each bin, we found no significant enhancement in isoprene during episodes of high exposure to the exhaust plume (Figure S2b). This is also confirmed by pairwise *t*-test between all exposure bins returning p-values > 0.05. We therefore discard the hypothesis of ship emissions significantly affecting the observed isoprene abundances.

Correlation Analysis

Correlation metrics between observed isoprene and chlorophyll-a were investigated using different approaches: firstly, chlorophyll-a along the ship track from the 8-day composite retrieval from MODIS-AQUA corresponding to the time of passage of the ACE vessel over a specific region of the SO. Secondly, we investigated whether introducing a lag between the observed isoprene and chlorophyll-a (i.e., if chlorophyll-a from the previous 8-day composite) led to an improved correlation. Lastly, we averaged chlorophyll-a along the back trajectory for each isoprene data point along the ship track. The results are shown in Table S1 below. The data in the table indicates that ambient isoprene exhibits the strongest correlation to chlorophyll-a during Leg 1 under all scenarios, and typically data north of 50°S exhibit higher correlation than data south of 50°S. Averaging along the back-trajectory does not improve the correlations, whereas introducing a lag time leads to a marginal improvement in the correlation.

Supplementary Tables

**Chlorophyll-a**	**Data subset**	**R^2**	**p-value**
MODIS 8-day composite, no lag, along cruise track only	All ACE	0.05	2e-09
	Leg 1	0.45	< 2.2e-16
	Leg 2	0.005	0.17
	Leg 3	0.002	0.68
	North of 50°S	0.20	< 2.2e-16
	South of 50°S	0.007	0.12
MODIS 8-day composite, 8-day lag, along cruise track only	All ACE	0.13	< 2.2e-16
	Leg 1	0.46	< 2.2e-16
	Leg 2	0.005	0.19
	Leg 3	0.001	0.77
	North of 50°S	0.20	2e-15
	South of 50°S	0.02	0.03
MODIS 8-day composite, no lag, average along adjusted back-trajectory	All ACE	0.02	3e-05
	Leg 1	0.25	< 2.2e-16
	Leg 2	0.004	0.15
	Leg 3	0.003	0.64
	North of 50°S	0.10	2e-11
	South of 50°S	0.007	0.07

Table S1. Correlation analysis between atmospheric isoprene observation and satellite retrievals of chlorophyll-a.

Supplementary References

1. Thurnherr, I. *et al.* Meridional and vertical variations of the water vapour isotopic composition in the marine boundary layer over the Atlantic and Southern Ocean. *Atmospheric Chem. Phys.* **20**, 5811–5835 (2020).
2. Bryant, D. J. *et al.* Biogenic and anthropogenic sources of isoprene and monoterpenes and their secondary organic aerosol in Delhi, India. *Atmospheric Chem. Phys.* **23**, 61–83 (2023).
3. Wang, N., Ernle, L., Bekö, G., Wargocki, P. & Williams, J. Emission Rates of Volatile Organic Compounds from Humans. *Environ. Sci. Technol.* **56**, 4838–4848 (2022).
4. Wohl, C. et al. Underway seawater and atmospheric measurements of volatile organic compounds in the Southern Ocean. Biogeosciences 17, 2593–2619 (2020).
